# Coherence in defect evolution data for the ion beam irradiated graphene

**DOI:** 10.1038/s41598-018-32300-w

**Published:** 2018-09-18

**Authors:** Sunmog Yeo, Jiyoon Han, Sukang Bae, Dong Su Lee

**Affiliations:** 1 Korea Multi-purpose Accelerator Complex, Korea Atomic Energy Research Institute, Gyeongju, Gyeongbuk 38180 Republic of Korea; 2 Institute of Advanced Composite Materials, Korea Institute of Science and Technology, Wanju-gun, Jeonbuk 55324 Republic of Korea

## Abstract

The defect evolution in graphene produced by ion beam bombardment is investigated by changing the ion species, irradiation energy and dose. Raman spectroscopy is performed to examine the defect yield produced under various ion beam bombardment conditions. The defect yields of the vacancy-type defect are well described by the linear energy transfer (*L*) and dose (*d*). By increasing *Ld*, the defect yields exhibit similar behaviours for all ion species. As a consequence, all the defect yields can be collapsed into a single curve by multiplying them by a single parameter, suggesting that the defect evolution under various ion beam bombardment conditions can be described in a simple formula.

## Introduction

Structural defects in graphene break the symmetry of the carbon triangular lattice and significantly alter its properties, usually in a destructive way. In other words, the well-known excellent electrical and mechanical properties of graphene are substantially lowered by introducing defects. However, it has been suggested that when the defects are controlled, they can give new functionalities to graphene. For instance, an extended line defect can act as a conducting wire in graphene-based electronic devices^1,2^. In addition, the sensitivity of graphene-based chemical gas sensors and the catalytic reaction can be enhanced by the adsorption energy reduction at the defect sites^3–8^ and Moreover, a theoretical study suggested that the defects at the polycrystalline boundary of graphene can create a tuneable transport gap^9^, which is essential for graphene nanoelectronic applications; such defects have been proven to be controllable via Joule heating^10,11^.

Raman spectroscopy has been widely used to characterize graphene^12,13^. The Raman D mode near 1350 cm^−1^ is particularly important for the defect characterization since it is related to the double resonance process mediated by an inelastic scattering with defects^14^. The intensity of the D peak, *I*(D), has been known as a measure of the number of defects when it is normalized by the Raman G peak intensity, *I*(G). The G mode is the only first-order phonon mode among the Raman signals of graphene^15^. In 1970, Tuinstra and König showed that the ratio *I*(D)/*I*(G) is related to the crystallite size (*L*_*a*_) of 3-dimensional graphite^16^. It is, however, notable that *I*(D)/*I*(G) cannot solely quantify the number of defects because the resonance condition changes for different incident laser energies. Recently, this ratio was better interpreted by Cancado *et al*. as *I*(D)/*I*(G) = 560/(*L*_*a*_·*E*_*L*_^4^), where *E*_*L*_^4^ is the laser excitation energy in eV^17,18^. On the other hand, defects in graphene are categorized into two types: vacancy type and sp^3^ type^15^. The vacancy-type defects are usually produced via ion bombardment, while the sp^3^-type defects are introduced via fluorination, hydrogenation and mild oxidation. Technically, Raman spectroscopy distinguishes the vacancy defects from the sp^3^ defects by comparing the intensity of the Raman D peak and D’ peak near 1620 cm^–1^; *I*(D)/*I*(D’) ~7 and 13 for the vacancy-type defects and the sp^3^-type defects, respectively.

Ion bombardment is a useful tool not only for nanopatterning of graphene^19,20^ but also for producing defects in graphene in a controlled manner. The density of holey defects is controlled by the ionic dose as long as the dose is not enough to merge the nearest defects^21^. In principle, the energy transfer between energetic ions and matters occurs via elastic collisions with the nuclei and inelastic collisions with the electron. For a low bombardment energy below ~100 keV, the nuclear collision is the origin of the defect formation in graphene^22,23^, while for a high bombardment energy, the electronic collision cannot be ignored^24,25^.

Although ion bombardment has been widely used, to our knowledge, systematic study on the defect evolution in graphene that combines the effects of different ion species, energies and doses is lacking. In this paper, we present the energy-dependent defect evolution using various ion species and doses. With a bombardment energy on the order of keV, the nuclear collision governs the defect formation in graphene^22,23^; hence, the nuclear term of the linear energy transfer (LET; in other words, the stopping power) is one of the crucial parameters for describing the number of defects in graphene^23^. Combining the LET and the ion dose, we successfully fit all the measured defect yields using a formula.

## Results

**Figure 1:**
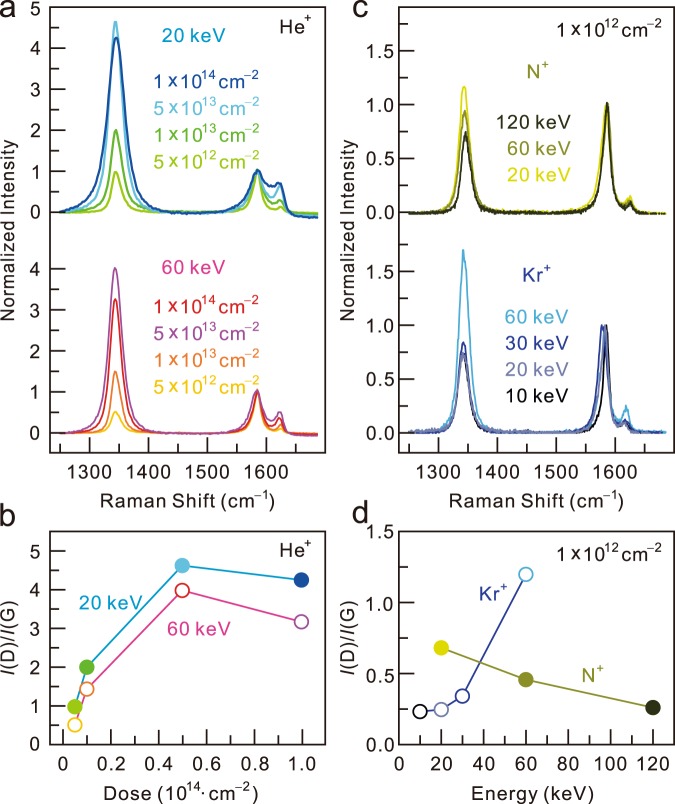
(**a**) Raman spectra of graphene obtained after He^+^ ion irradiation for various doses (5 × 10^12^, 1 × 10^13^, 5 × 10^13^ and 1 × 10^14^ ions/cm^2^) at energies of 20 (top) and 60 (bottom) keV. (**b**) *I*(D)/*I*(G) for He^+^ ion irradiation as a function of dose. (**c**) Raman spectra of graphene after N^+^ (top) and Kr^+^ ion irradiation (bottom) for various energies. (**d**) *I*(D)/*I*(G) for N^+^ and Kr^+^ ion irradiation as a function of energy.

Figure 1(a) shows Raman spectra of graphene after He^+^ irradiation with a typical evolution of the defect-induced peak at ~1340 cm^−1^ corresponding to an increase in irradiation from 5 × 10^12^ to 1 × 10^14^ ions/cm^2^ at energies of 20 (upper panel) and 60 keV (lower panel). To compare the number of defects under the various irradiation conditions, the Raman data were normalized by *I*(G). The D peak of pristine CVD graphene was typically very low and *I*(D)/*I*(G) = 0.028 ± 0.013 (data not shown). The normalized intensity at an energy of 20 keV increases with increasing He^+^ ion irradiation dose up to 5 × 10^13^ ions/cm^2^. At larger doses, *I*(D)/*I*(G) would reach to the maximum and begin to decrease showing amorphous phase exhibiting a large bump near D and G modes in Raman data^12^. The scattering of the electron-hole pairs with optical phonons in the double resonance process is suppressed due to the very short inter-defect distance^21,26^. In this paper, we exclude these amorphous phases. Defects in graphene are categorized into two types: vacancy type and sp^3^ type^14^. The vacancy-type defects are usually produced via ion bombardment, while the sp^3^-type defects are introduced via fluorination, hydrogenation and mild oxidation. Technically, Raman spectroscopy distinguishes the vacancy defects from the sp^3^ defects by comparing the intensity of the Raman D peak and D’ peak near 1620 cm^-1^; *I*(D)/*I*(D’) ~7 and 13 for the vacancy-type defects and the sp^3^-type defects, respectively^15^. Here, we focus only on the vacancy-type defects. We tested the *I*(D)/*I*(D’) values of all measured Raman data and selected only the data that satisfy the criterion *I*(D)/*I*(D’) ~7 for further analysis. At a dose of 5 × 10^13^ ions/cm^2^, the inter-defect distance (*l*) is ~1.5 nm calculated by $l=1/\sqrt{d}$ with the assumption of point defects. The value can be an overestimation considering collision cascade effect which can occur in high energy ion bombardment^21,27^. It is worth noting that the maximum *I*(D) was previously observed at the inter-defect distance of ~3 nm for low energy (90 eV) Ar^+^ bombardment^21^. This implies that the ion dose required to obtain the maximum *I*(D)/*I*(G) depends on the ion species^28,29^. Moreover, the *I*(D)/*I*(G) also differs for different irradiation energies. For an irradiation energy of 60 keV, the normalized intensity exhibits a similar tendency, but the values are smaller than those for 20 keV, as shown in Fig. 1(b).

Figure 1(c,d) show the energy dependence of the normalized intensity for the N^+^ and Kr^+^ ion at a fixed dose of 1 × 10^12^ ions/cm^2^. *I*(D)/*I*(G) monotonically decreases with increasing N^+^ ion irradiation energy, while for Kr^+^ ion irradiation, it increases with increasing irradiation energy up to 60 keV. This means that the energy-dependent behaviour of *I*(D)/*I*(G) is affected by the ion species. From measurements under various conditions and ion species, we conclude that the defect yield is dependent not only on the ion dose but also on the ion species and its irradiation energy. Basically, it is clear that the defect yield is proportional to the ion beam dose. However, the influence of the irradiation energy and ion species on the defect yield is somewhat complex at first sight. When an energetic ion passes through a matter, the ion loses its energy via scattering with electrons and nuclei in the matter. To consistently describe the defect yield under the various ion irradiations, a linear energy transfer (LET) parameter is useful, where the LET is the energy loss per unit length of an energetic ion, LET = −*dE*/*dz*, where *z* is the distance in the direction of irradiation. In principle, the LET consists of two parts: nuclear LET and electronic LET. Since the defects in graphene are formed by nuclear collisions between carbons and irradiated ions with energies below ~120 keV, the electronic LET is ignored^25^.

**Figure 2:**
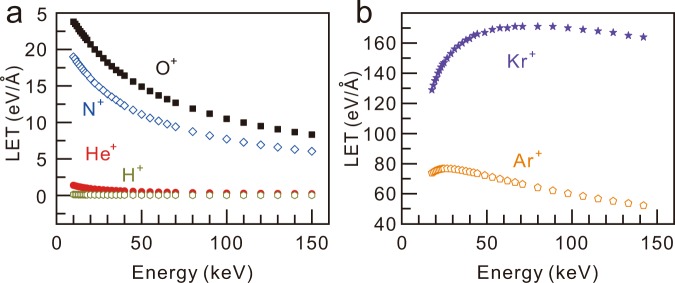
LET as a function of energy for various ion species: H^+^, He^+^, N^+^, and O^+^ (**a**) and Ar^+^ and Kr^+^ (**b**).

We calculated the LET for all the ions irradiated in the experiments using the SRIM (Stopping and Range of Ions in Matter) code. Figure 2 shows the SRIM calculation results of the LET as a function of energy for various ions. For the calculation, the stopping matter was set as a real structure with 0.345 nm-thick graphene and a SiO_2_ layer. Light ions, i.e., He^+^, H^+^ and O^+^, exhibit monotonically decreasing LET curves with increasing *E*. Heavier ions exhibit non-monotonous behaviours. The LET for the Ar^+^ ion increases for *E* < 20 keV but decreases at higher energies. For Kr ions, the maximum LET appears at ~65 keV. The calculated LET results qualitatively explain the discrepancy in the energy dependence of *I*(D)/*I*(G) for N^+^ and Kr^+^ ions shown in Fig. 1(d). The defect yield for N^+^ ion irradiation monotonically decreases with increasing *E*, which is in agreement with the LET calculation. Additionally, the monotonous increase in *I*(D)/*I*(G) for the Kr^+^ ion as *E* increases to 60 keV can be similarly explained by the LET curve for the Kr^+^ ion. The similarity between the behaviours of *I*(D)/*I*(G) and the LET implies that the defect yield is related to the LET of the irradiation ions.

## Discussions

**Figure 3:**
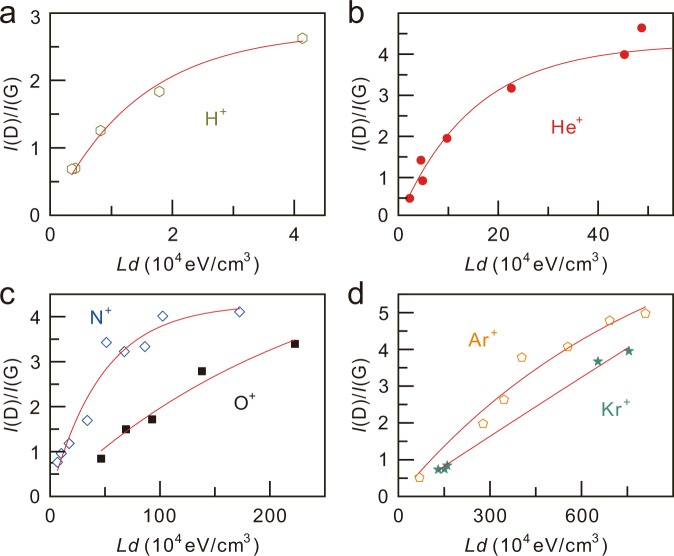
*I*(D)/*I*(G) as a function of *L·d* for various ion species: H^+^ (**a**), He^+^ (**b**), N^+^, and O^+^ (**c**) and Ar^+^ and Kr^+^ (**d**). The red lines are the fittings.

To understand the relation between the defect yield and the LET, we performed a calculation of the defective area as a function of the irradiation dose. For simplicity, we assumed that a point defect is generated by the irradiation of an ion and that the resulting defective area is a circle with a radius *r*. The defective area increases as a result of additional irradiation of an ion, i.e., *A*_*n+1*_ − *A*_*n*_ = *πr*^2^ (*A*_t_ − *A*_*n*_)/*A*_t_, where *A*_*n*_ and *A*_t_ are the defective area after irradiation of the *n*^th^ ion and the total area of graphene (*A*_t_ = *πR*^2^), respectively. With the initial condition of *A*_0_ = 0, we obtain ${A}_{n}=1-{(1-\gamma )}^{n}$, where *γ* = *r*^2^/*R*^2^. The ion irradiation dose can be expressed as *d* = *n*/(*πR*^2^); then, ${A}_{n}=1-{(1-\gamma )}^{d\pi {R}^{2}}=1-{(1-\gamma )}^{\frac{1}{-\gamma }\cdot (-d\pi {r}^{2})}$. When the dose is large enough and *r*/*R* ≪ 1, we can estimate the area as follows: $A(d,\,r)\approx 1-{e}^{-\pi {r}^{2}d}$. Here, we assume that the defective area (*πr*^2^) is proportional to the LET (*L*), as the energy transferred from the accelerated ions should be positively correlated with the defect creation, i.e., *I*(D)/*I*(G) = *C*(1 − *e*^–*αLd*^). Figure 3 displays the defect yields, *I*(D)/*I*(G) as a function of *L·d* for various ion species, and the fitting curves, using *C* and *α* as fitting parameters. The coefficients of determination for all the fittings were larger than 0.96, indicating that the equation including the LET and dose well explains both the energy and dose dependent behaviours of *I*(D)/*I*(G).

**Figure 4:**
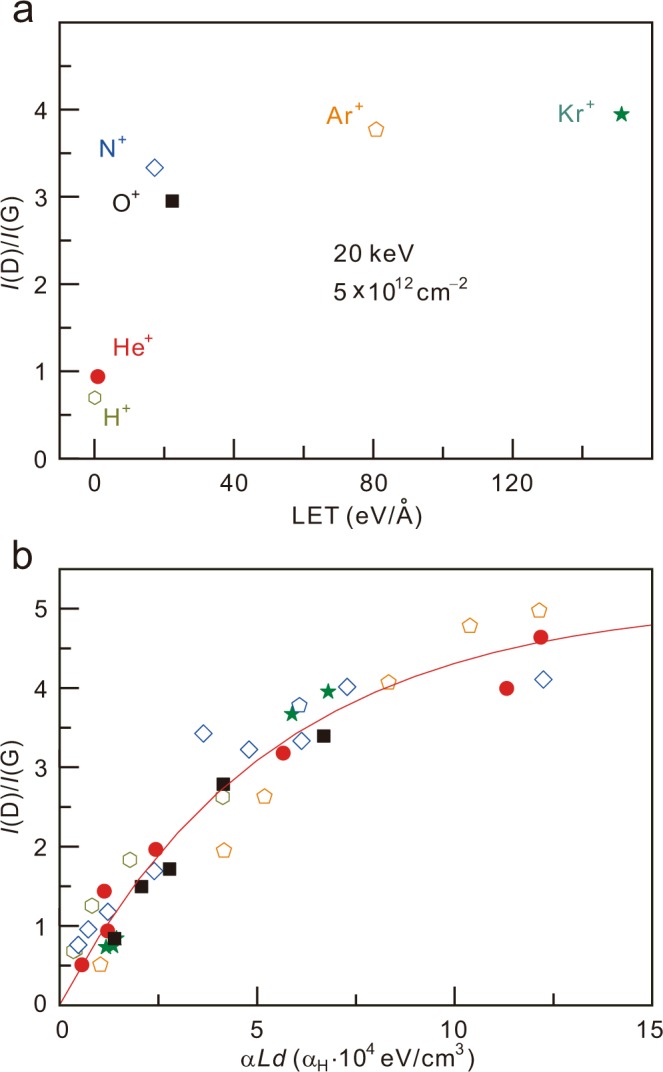
(**a**) *I*(D)/*I*(G) as a function of the LET for different ion species and the same energy and dose. (**b**) All *I*(D)/*I*(G) data points as a function of *α·L·d*.

The defect yields for different ions at the same irradiation energy of 20 keV and dose of 5 × 10^12^ ions/cm^2^ are plotted as a function of the LET in Fig. 4(a). The heavier ions generate more defects due to their larger LET. Interestingly, the fitting curves can be described in a universal form by regulating a parameter *α*, which varies for different ion species because another fitting parameter *C* (~5.1) does not significantly vary with the ion species. We obtain the *α* values for the ions relative to the parameter for the H^+^ ion (*α*_H_) as *α*_He_ ~ 0.250·*α*_H_, *α*_N_ ~ 0.0714·*α*_H_, *α*_O_ ~ 0.313·*α*_H_, *α*_Ar_ ~ 0.0149·*α*_H_ and *α*_Kr_ ~0.00901·*α*_H_. Note that the parameter *α* is roughly proportional to the inverse mass number. Although we describe a simple formula using the LET, other ways to express the relation may exist. To exactly describe the coherence in defect evolution, a few things should be additionally considered. First, the collision between irradiated ions and carbon atoms in the graphene lattice should be precisely simulated, as the graphene lattice is truly two-dimensional and the LET does not reflect the exact amount of energy that the ions transfer to the graphene lattice. Additionally, the back scatterings from the silicon oxide beneath the graphene may need to be considered^30,31^. These may more precisely explain the bombardment ion dependence of *α* and the correlated formula of the Raman defect yield, which involves not only the ion irradiation dose but also the ion kinetic energy and ion species.

In conclusion, the defect evolution in graphene is investigated by means of ion beam irradiation under various conditions, i.e., ion dose, irradiation energy and ion species. The vacancy-type defects induced by the nuclear contribution are analysed based on the ion dose (*d*) and the linear energy transfer (*L*) estimated using the SRIM code. The defect yield and the ratio between Raman D and G peaks are plotted as functions of *Ld* and fitted using the equation *I*(D)/*I*(G) = *C*(1 − *e*^–*αLd*^). Furthermore, by multiplying a single parameter (*α*) by *Ld*, all the data collapse into a single curve, suggesting that a simple formula govern the defect evolution initiated by ion beam irradiation.

## Methods

### Graphene sample preparation

Samples were prepared by transferring a graphene layer from a Cu foil to a Si substrate with a 300 nm-thick oxide layer on top. The graphene layers used for the experiment were synthesized via the chemical vapor deposition (CVD) method at 1000 °C with hydrocarbon source (CH_4_, 10 sccm) and hydrogen (H_2_, 100 sccm) gas flow.

### Ion beam irradiation

H^+^, He^+^, N^+^, O^+^, Ar^+^ and Kr^+^ ions were produced using a duoPIGatron ion source, and their species were separated using a mass separation magnet. The selected ions were accelerated in an electrostatic acceleration tube up to 120 keV and irradiated onto the graphene sample. The pressure in the target chamber was below 5 × 10^−6^ Torr. The ion dose was measured using a Faraday cup in conjunction with a Tektronix oscilloscope. In order to minimize the sputtering effects, all the ion beams were irradiated on the perpendicular to the samples.

### Raman spectroscopy

A Raman experiment was performed using a Horiba LabRAM HR system under ambient conditions using a laser with a wavelength of 514 nm. We used a low incident laser power less than 0.5 mW with a spot size of ~1 μm and a short irradiation time of 10 sec to prevent any additional degradation of the graphene by the laser irradiation during the Raman measurement.
